# Crohn’s disease recurrence updates: first surgery *vs*. surgical relapse patients display different profiles of ileal microbiota and systemic microbial-associated inflammatory factors

**DOI:** 10.3389/fimmu.2022.886468

**Published:** 2022-07-29

**Authors:** Edda Russo, Lorenzo Cinci, Leandro Di Gloria, Simone Baldi, Mario D’Ambrosio, Giulia Nannini, Elisabetta Bigagli, Lavinia Curini, Marco Pallecchi, Donato Andrea Arcese, Stefano Scaringi, Cecilia Malentacchi, Gianluca Bartolucci, Matteo Ramazzotti, Cristina Luceri, Amedeo Amedei, Francesco Giudici

**Affiliations:** ^1^ Department of Experimental and Clinical Medicine, University of Florence, Florence, Italy; ^2^ Department of Neurosciences, Psychology, Drug Research and Child Health (NEUROFARBA), University of Florence, Florence, Italy; ^3^ Department of Biomedical, Experimental and Clinical Sciences “Mario Serio”, University of Florence, Florence, Italy; ^4^ Enteric Neuroscience Program, Department of Medicine, Section of Gastroenterology and Hepatology, Mayo Clinic, Rochester MN, United States

**Keywords:** Crohn’s disease, recurrence, microbiota, miRNA, free fatty acids, SCFA

## Abstract

**Background and aims:**

Crohn’s disease (CD) pathogenesis is still unclear. Remodeling in mucosal microbiota and systemic immunoregulation may represent an important component in tissue injury. Here, we aim to characterize the ileal microbiota in both pathological and healthy settings and to evaluate the correlated systemic microbial-associated inflammatory markers comparing first-time surgery and relapse clinical conditions.

**Methods:**

We enrolled 28 CD patients at surgery; we collected inflamed and non-inflamed mucosa tissues and blood samples from each patient. Bacterial wall adherence was observed histologically, while its composition was assessed through amplicon sequencing of the 16S rRNA gene. In addition, we evaluated the systemic microRNA (miRNA) using quantitative real-time PCR amplification and free fatty acids (FFAs) using gas chromatography–mass spectroscopy.

**Results:**

The total number of mucosal adherent microbiota was enriched in healthy compared to inflamed mucosa. In contrast, the phylum *Tenericutes*, the family *Ruminococcaceae*, and the genera *Mesoplasma* and *Mycoplasma* were significantly enriched in the pathological setting. Significant microbiota differences were observed between the relapse and first surgery patients regarding the families *Bacillaceae 2* and *Brucellaceae* and the genera *Escherichia/Shigella*, *Finegoldia*, *Antrobacter*, *Gemmatimonas*, *Moraxella*, *Anoxibacillus*, and *Proteus*. At the systemic level, we observed a significant downregulation of circulating miR-155 and miR-223, as well as 2-methyl butyric, isobutyric, and hexanoic (caproic) acids in recurrence compared to the first surgery patients. In addition, the level of hexanoic acid seems to act as a predictor of recurrence risk in CD patients (OR 18; 95% confidence interval 1.24–261.81; *p* = 0.006).

**Conclusions:**

We describe a dissimilarity of ileal microbiota composition comparing CD and healthy settings, as well as systemic microbial-associated inflammatory factors between first surgery and surgical relapse. We suggest that patterns of microbiota, associated with healthy ileal tissue, could be involved in triggering CD recurrence. Our findings may provide insight into the dynamics of the gut microbiota–immunity axis in CD surgical recurrence, paving the way for new diagnostics and therapeutics aimed not only at reducing inflammation but also at maintaining a general state of eubiosis in healthy tissue.

## Introduction

Inflammatory bowel disease (IBD), which includes Crohn’s disease (CD) and ulcerative colitis (UC), is defined by intermittent chronic inflammation of the gastrointestinal system, resulting in bowel damage. CD, a multifactorial disorder that causes significant life-long impairment, commonly begins in young adulthood and is accompanied by periods of remission and recurrence (1). Nevertheless, surgical recurrence, or the necessity for a further operation, has been reported in 25% to 45% of patients within 10 years following the initial bowel resection (2). The transmural and segmental inflammation in CD is typically concentrated in the terminal ileum, but the pathophysiology remains unknown. Moreover, the drivers of recurrence following ileocolonic resection (ICR) remain hypothetical and challenging.

Current data have suggested that a complex interplay of genetic, epigenetic, microbial, metabolic, and environmental factors promotes, at the gut level, aberrant innate immune responses, as reviewed in Zheng et al. (3). In fact, mucosal immunoregulation dysfunctions may play a role in the etiology of chronic intestinal inflammation and tissue damage (4). Moreover, several reports have shown that the multifaceted regulatory mechanisms linked with mucosal immunity against microbial flora, epithelial barrier dysfunction, and environmental influences can cause an abnormal inflammatory response, contributing to CD pathogenesis (5–7). Furthermore, in CD patients, the richness of mucosal microbiota (and its metabolites) was strongly linked with disease activity or exacerbations (8).

Increasing data point to the importance of gut microbiota (GM) alterations in CD pathogenesis (9), involving decreased abundance of *Bacteroides*, *Firmicutes*, *Clostridia*, and *Lactobacillus*, *Ruminococcaceae* and increased abundance of *Gammaproteobacteria* and *Enterobacteriaceae* (10). Some studies have examined the gut microflora at surgery to find microbial profiles associated with remission or recurrence (11). It has been observed that ileal mucosa–associated microflora changed significantly after surgery, including variations between patients with and without recurrence (12, 13). Through a comprehensive analysis of ileal tissue layers, inflammatory response, and microbiota composition, we have recently reported that the phylum *Tenericutes* and the genera *Mesoplasma* and *Mycoplasma* were significantly augmented in the inflamed tissues (14). Moreover, we reported different and significant cytokine levels at the three tissue layers (mucosa, submucosa, and serosa) and differences in bacterial flora composition between the relapse and first surgery patients (14).

Other key factors acting as regulators of inflammatory responses and metabolic pathways are free fatty acids (FFAs), classified into short–chain fatty acids (SCFAs), medium–chain fatty acids (MCFAs), and long–chain fatty acids (LCFAs). SCFAs are produced as a result of bacterial and host metabolism. Recent studies have shown that SCFAs and LCFAs play a vital role in CD pathophysiology and development by various mechanisms, i) affecting pro– and anti–inflammatory mediators, ii) maintaining intestinal homeostasis, and iii) regulating gene expression. In particular, SCFAs and LCFAs activate signaling cascades that control immune functions through interaction with cell surface free fatty acid receptor, while SCFAs are crucial to maintaining the host’s normal gut physiology and metabolic functions and that a part of them enters the systemic circulation (15).

Additionally, several studies suggest that the GM may interact also with microRNAs (miRNAs) in regulating host pathophysiology and many immune processes. However, how miRNA circuits orchestrate aberrant intestinal inflammation during inflammatory CD and recurrence phenomena is poorly defined. Furthermore, miRNAs can regulate bacterial composition by specifically targeting bacterial genes, and they can be used as markers of microbial fluctuations in intestinal pathologies (16). In addition, the GM has been found to regulate the host miRNA expression, primarily through the GM metabolites, such as lipopolysaccharide (LPS) and SCFAs such as butyrate (17, 18). Specific miRNAs are related to microbiome and inflammatory processes, including miR–223 and miR–155 (19). Chief among these, miR–223 is emerging as an important regulator of the innate immune system and the response to bacterial stimulation (20, 21), the upregulation of miR–223 is reported as a novel biomarker in subsets of IBD patients (22) as well as in preclinical models of intestinal inflammation (19, 23). miR–155 influences numerous biological functions including inflammatory response, intracellular signaling cascades, regulation of cytokine production, and response to microbiota (24–27). miR–423 is also a pro–inflammatory miRNA, tightly controlled by IL–21 in IBD. It directly targets claudin–5, a critical family member in the maintenance of normal intestinal barrier property, and regulates the NF–κB/MAPK/JNK signaling pathway (28).

This emerging evidence suggests that “taking an instant picture” of all the above mentioned microbial, metabolic, and epigenetic factors, involved in the intricate transmural and systemic inflammatory process, might be useful to deepen our understanding of the CD recurrence dynamics.

Starting from these premises, through a holistic and innovative analysis of ileal microbiota composition, circulating FFAs, and miRNAs, we investigated the mutual dialogue between CD microbial and inflammatory factors, comparing healthy and pathological tissues as well as first surgery and relapse conditions.

Our results showed a different regulation of the gut microbiota–immunity axis, corroborating our previous results (14) and expanding them with new details and perspectives on circulating microbiota–related factors (SCFAs and miRNAs), at first surgery time and relapse conditions. Moreover, we observed a potential involvement of microbiome patterns, associated to healthy ileal tissue, in triggering recurrence–linked determinants, suggesting that not only anti–inflammatory therapies but also GM eubiosis maintenance may be crucial for early treatment adjustments.

## Materials and methods

### Patients

We enrolled 28 adult patients (16 men and 12 women, mean age 46.82 years, age range 19–72 years) affected by ileal CD (**Table 1**) at “Careggi University Hospital” (Florence, Italy) between 2018 and 2019, after obtaining informed consent and approval of the local ethics committee (study no. 2016.0842). Among these 28 patients, the clinical and anamnestic data, as well as the ileal microbiome sequences from 10 patients, were previously included in a study already published by our group (14) describing the interplay between ileal cytokines at three different tissue layers (mucosa, submucosa, and serosa) and mucosal microbiota, in first surgery and relapse CD.

**Table 1:** Clinical features of the enrolled patients.

No.	Sex^a^	CD site^b^	Age of CD onset	Surgery	Disease behavior^c^	Smoking status^d^	Familiarity	BMI	Malnutrition according to ESPEN 2015	CD duration (years)	Age at surgery (years)	Period from previous surgery (years)	Comorbidities^e^	CD therapies
1	M	L2	27	First surgery	B2	No	No	22.4	No	4	31	–	–	Infliximab, methylprednisolone
2	M	L2	23	First surgery	B2, B4	No	No	17.4	Yes	11	27	–	–	Methylprednisolone
3	M	L2	18	First surgery	B2, B3	No	No	20.7	No	1	19	–	–	–
4	M	L2	11	First surgery	B2, B3	No	No	22.1	No	1	30	–	–	Adalimumab
5	M	L2	16	Relapse	B3	Cur	No	16.8	Yes	29	45	4	–	Azathioprine, vedolizumab
6	M	L1	37	First surgery	B2	Ex	No	32.8	No	13	50	–	BS, H, OSAS	Mesalamine, omeprazole, prednisone
7	M	L1	17	Relapse	B3	Ex	No	20.1	No	23	40	9	O	Risedronate
8	F	L2	40	First surgery	B2	No	No	30.5	No	1.5	41	–	A, AS	Beclomethasone
9	F	L1	63	First surgery	B2	No	No	24.2	No	7	70	–	–	Mesalamine, sulfasalazine
10	F	L2	40	First surgery	B2, B3	No	No	18.7	No	10	50	–	–	Mesalamine
11	M	L2	24	Relapse	B2	No	No	23.3	No	16	40	16	–	Mesalamine
12	M	L2	28	Relapse	B2, B3	No	No	27.9	No	33	61	19	AS	–
13	F	L2	37	Relapse	B2	No	No	21.6	No	12	49	12	–	Prednisone
14	M	L2	40	Relapse	B2	No	No	20.1	No	12	53	0.5	–	Mesalamine
15	F	L2	42	Relapse	B2	No	No	16.6	Yes	29	71	21	MD	Prednisone
16	F	L2	64	First surgery	B2	Yes	Yes	21.3	Yes	5	69	–	H	–
17	M	L1	62	First surgery	B2	No	No	23.9	Yes	2	64	–	DM, GA, HHG	Allopurinol, budesonide, mesalamine
18	F	L2	21	Relapse	B3, B4	No	No	23.5	No	36	57	36	EA, H	Mesalamine, ranitidine
19	M	L1	36	First surgery	B2	Ex	No	22,2	No	4	40	–	–	Budesonide
20	F	L1	17	Relapse	B2, B3	No	No	21	No	1	17	1	–	Budesonide
21	M	L2	47	First surgery	B2	No	No	20.9	No	12	60	12	–	Mesalamine, prednisone
22	F	L2	29	First surgery	B2	Yes	No	22.8	No	28	57	–	–	–
23	F	L2	32	Relapse	B2	Yes	No	21.5	No	13	45	13	–	–
24	F	L2	21	First surgery	B2	No	No	20	No	1 month	21	–	–	Methylprednisolone, piperacillin/tazobactam
25	M	L1	13	First surgery	B2	Yes	No	27.8	No	13	26	–	HZ	Adalimumab
26	M	L1	49	First surgery	B2	Yes	No	21.1	No	5	54	–	P	–
27	M	L1	37	First surgery	B3	No	No	19.1	No	6	43	–	–	Mesalamine
28	M	L1	34	Relapse	B3	Yes	No	18.8	No	13	47	13	–	Prednisone

^a^Sex: M, male, F, female. ^b^CD site: L1, terminal ileum, L2, ileum colon. ^c^Disease behavior: B1, non–stricturing, non–penetrating, B2, stricturing, B3, penetrating, B4, perianal disease. ^d^Smoking status: No, non–smoker, Ex, ex–smoker, Cur, current smoker. ^e^Comorbidities: A, asthma, AS, ankylosing spondylitis, BS, Brown–Sequard syndrome, DM, Dupuytren’s morbus, EA, enteropathic arthritis, GA, gouty arthritis, H, hypertension, MD, major depression, NHG, non–Hodgkin lymphoma, O, osteoporosis, OSAS, obstructive sleep apnea syndrome, HZ, herpes zoster, P, psoriasis.

As reported in our previous study (14), ileal CD diagnosis had been performed by clinical/endoscopic criteria and confirmed by histological analysis. The inclusion criteria were as follows: i) diagnosis of IBD for at least 3 months according to the standard Montreal classification (29), ii) patients with IBD with ileocolic localization, and iii) patients aged 25–70 years. The exclusion criteria include the following: i) use of antibiotics or any other probiotic bacterial supplement in the previous 1 month, ii) use of non–steroidal anti–inflammatory drugs (NSAIDs) in the previous 1 month, iii) reported recent diagnosis (less than 3 months) of bacterial or parasitic infections of the gastrointestinal tract, and iv) trip to exotic areas in the last 5 years. Patients have not been treated with antibiotics, aminosalicylates or immunosuppressants, biological therapy, and steroids for at least 3 weeks before the ICR.

### Bacterial wall adherence

Samples of affected (based on visual expert inspection) and apparently normal mucosa, harvested at surgery, were fixed in Carnoy’s solution and embedded in paraffin. Sections (4 µm) were stained with 2.5 ng/ml of ethidium bromide and observed with an Olympus BX63 microscope equipped with a metal halide lamp (Prior Scientific Instruments Ltd., Cambridge, United Kingdom) and a digital camera (Olympus XM10, Olympus, Milan, Italy).

Ten photomicrographs were randomly taken for each section at ×400 magnification, and the number of adherent bacteria was evaluated using ImageJ 1.33 image analysis software (http://rsb.info.nih.gov/ij).

### Characterization of paraffin–embedded tissue microbiota

The microbiota of paraffin–embedded tissue was assessed as described in our previous study (14). In detail, for each sample, the first few scrolls from the paraffin–embedded blocks were discarded and then eight 10–μm scrolls were cut and placed into sterile 2–ml centrifuge tubes. For deparaffinization, we used xylene (Sigma–Aldrich, MO, USA) for all extractions. DNA was extracted using the QIAamp DNA FFPE tissue kit (Qiagen, CA, USA) according to the manufacturer’s protocol. Nucleic acids were eluted in a 50–μl elution buffer following a 5–min column incubation at room temperature. The microtome was cleaned with DNA AWAY (Thermo Scientific, MA, USA) between each sample, and the equipment was regularly tested using sterile swabs that also underwent sequencing as controls. The quality and quantity of extracted DNA were assessed using the NanoDrop ND–1000 (Thermo Fisher Scientific, Waltham, MA, USA) and the Qubit Fluorometer (Thermo Fisher Scientific), respectively. Then, genomic DNA was frozen at −20°C.

The extracted DNA samples were sent to IGA Technology Services (Udine, Italy) where amplicons of the variable V3–V4 region of the bacterial 16S rRNA gene were sequenced in paired–end (2 × 300 cycles) on the Illumina MiSeq platform, according to the Illumina 16S rRNA amplicon.

The bioinformatic analysis of ileal microbiome sequences was performed following the methods already described in our previous studies (14, 30). In detail, regarding the sequencing library preparation protocol (31), raw sequences were processed following the software pipeline MICCA (32). Paired–end reads were assembled using the “mergepairs” command, maintaining a minimum overlap of 100 bp and an edit distance in the maximum overlap of 32 bp. Subsequently, the sequences were cut with the “trim” command in order to remove the primers and eventually eliminate the reads with imperfections in primer sequences. All the reads with a length lower than 350 bp and with an error rate higher than or equal to 0.5 were removed with the “filter” command. The cleaned reads were eventually merged into a single file with the “merge” command and transformed into a fasta file. The operational taxonomic units (OTUs) were generated using the “out” command in “denovo_greedy” mode, setting a 97% identity and performing an automatic removal of chimeras with the “–c” option. The longest sequence of each OTU was used for the taxonomic assignment using the “classify” command in “rdp” mode, i.e., using the RDP Bayesian classifier that is able to obtain classification and confidence for taxonomic ranks up to genus.

Statistical analyses on the bacterial community were performed in R (R Core Team, 2014) with the help of the packages phyloseq 1.26.1 (33), DESeq2 1.22.2 (34), breakaway 4.6.16 (35), and other packages satisfying their dependencies, particularly vegan 2.5–5 (35). Rarefaction analysis on OTUs was performed using the function rarecurve (step 50 reads), further processed to highlight saturated samples (arbitrarily defined as saturated samples with a final slope in the rarefaction curve with an increment in OTU number per reads <1e−5). For the cluster analysis (complete clustering on Euclidean distance) of the entire community, the OTU table was first normalized using the total OTU counts of each sample and then adjusted using square root transformation. The coverage was calculated by Good’s estimator using the formula: (1 − *n*/*N*) × 100, where *n* is the number of sequences found once in a sample (singletons), and *N* is the total number of sequences in that sample.

Richness, Shannon, Chao 1, and evenness indices were used to estimate bacterial diversity in each sample using the function estimate_richness from phyloseq (33). The evenness index was calculated using the formula *E* = *S*/log(*R*), where *S* is the Shannon diversity index and *R* is the number of OTUs in the sample. Differences in all indices between CD and healthy tissues were tested using a paired Wilcoxon signed–rank test. Sample richness was further measured using the estimator and its associated error was introduced in the breakaway package (35). The function betta_random of breakaway was further used to evaluate the statistical differences in richness between paired–by–patient samples. The differential analysis of abundance at the OTUs as well as at the different taxonomic ranks (created using the tax_glom function in phyloseq) was performed with DESeq2 (34) using two groups blocked by patient design in order to perform a paired test when needed (14).

### miRNA analyses

Total RNA was extracted from 200 µl of plasma by using TRIzol (Invitrogen, Life Technologies, Carlsbad, CA, USA), according to the instructions provided by the manufacturer.

For miRNA–specific cDNA synthesis, total RNA was reverse–transcribed using the miRCURY LNA RT kit (Qiagen). Quantitative real–time PCR amplification and analysis were performed using the Rotor–Gene Q thermal cycler (Qiagen, Hilden, Germany), the miRCURY LNA SYBR^®^ Green PCR kit, and the specific miRCURY LNA miRNA PCR Assay for miR–423, miR–223–3p, and miR–155–3p. RNU–6B was used as endogenous control and relative expression levels among miRNAs and RNU–6B were calculated using the 2^−dCT^ method.

### Evaluation of FFAs by GC–MS analysis

Analysis of the FFAs was performed using an Agilent GC–MS system composed of an HP 5971 single quadrupole mass spectrometer, an HP 5890 gas chromatograph, and an HP 7673 autosampler, according to previously described protocols (36). Briefly, just before the analysis, each sample was thawed and the FFAs were extracted as follows: an aliquot of 300 µl of serum sample was added to 10 μl of ISTD mixture, 100 μl of tert–butyl methyl ether, and 20 µl of 6 M HCl + 0.5 M NaCl solution in a 0.5–ml centrifuge tube. Afterward, each tube was stirred in a vortex for 2 min and centrifuged at 10,000 rpm for 5 min, and finally, the solvent layer was transferred to a vial with a microvolume insert and analyzed.

### Statistical analysis

The software GraphPad Prism 7.00 and Statgraphics Centurion XVI were used for statistical data analysis. Categorical variables were expressed as frequency with proportions and compared by chi–square or Fisher’s exact test, when necessary. Continuous data were presented as mean ± standard deviation (SD) or median with interquartile range (IQR) and compared using the Mann–Whitney/Kruskal–Wallis test or *t*–test/one–way ANOVA, depending on data distribution. Linear regression was applied to identify the correlation between two variables.

The odds ratio and 95% confidence intervals for recurrence risk were calculated using logistic regression analysis. *p <*0.05 was considered statistically significant.

Correlations between FFAs and taxonomies that varied significantly between the first operation and surgery relapse patients (both in CD tissue and healthy tissue) were evaluated using Spearman’s rank correlation analysis, under the assumption that there is a non–linear relationship between the examined variables. Correlations with adjusted *p*–value lower than 0.05 were considered significant.

## Results

### Microbiota analysis in CD *vs*. healthy ileal mucosa

#### Distinct bacterial wall adherence in CD *vs*. healthy ileal mucosa

**Figure 1:**
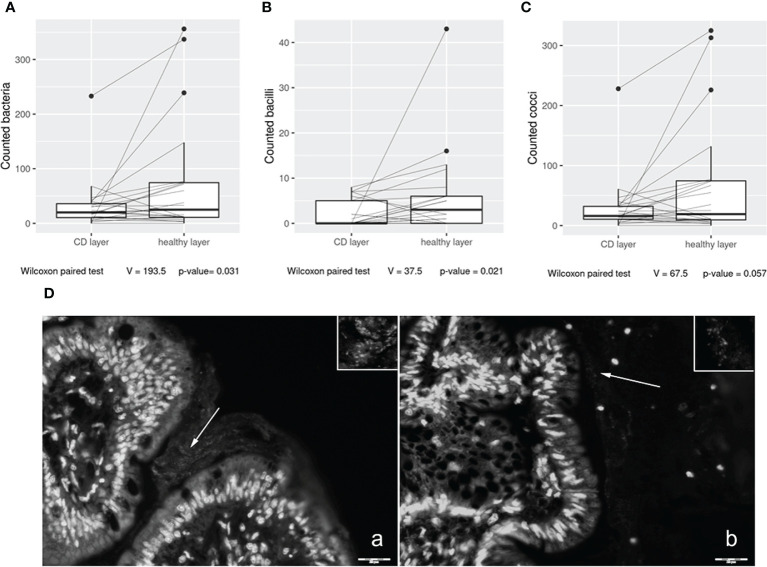
Box plot distribution of adherent bacteria counts **(A)**, bacilli **(B)**, and cocci **(C)** identified in healthy tissues and Crohn’s disease (CD) samples. Analyses were assessed using the paired Wilcoxon signed–rank test, and *p*–values less than 0.05 were considered statistically significant. Mucosa–associated bacteria in histological samples **(D)**. Images collected at ×400 magnification indicate **(A)** H mucosa sample and **(B)** CD sample. White arrows: bacteria present in the mucus contiguous to the mucosa. Insert magnification: bacteria. Scale bar: 20 µm.

In the first part of the study, we investigated bacterial wall adherence in healthy and CD mucosal paraffin–embedded tissues, collected at the time of ICR from 24 out of 28 patients. In detail, using a microscope, we evaluated the number of total adherent bacteria and we were able to perform a morphological distinction between cocci and bacilli. The number of observed total bacteria was enriched in healthy *vs*. CD tissues (*p* = 0.03) (**Figure 1**) suggesting a dysbiotic condition. Moreover, bacteria having bacillus morphology were significantly enriched in healthy *vs*. CD tissues (*p* = 0.021) (**Figure 1**), while a trend was observed in the number of cocci (*p* = 0.057) (**Figure 1**). As the most represented ileal bacteria having bacillus morphology are *lactobacilli* and *bifidobacteria*, known to be inflammation–suppressing taxa, their depletion in the CD setting suggests a loss of microbial defense against tissue inflammation. Representative images of bacterial wall adherence are shown in **Figure 1** (a and b).

#### Dysbiotic variation of the ileal microbiota

Since we detected significant total bacterial count variations between the affected and healthy mucosa tissues, we deeply characterized the resident ileal microbial communities of retrospectively diagnostic paraffin–embedded sections in 23 CD (of the 28 samples, 5 showed insufficient amount for analysis) patients using 16S rRNA amplicon sequencing.

We obtained a total of 4,051,315 reads, and after all the steps of preprocessing (pair merging, trimming, quality filtering, and chimera detection), a total of 2,325,681 (57.4%) were available for further analysis. The samples showed a Good’s coverage ranging from 99.90% to 99.98% indicating that less than 1% of the reads in a given sample came from OTUs that appear only once in that sample.

**Figure 2:**
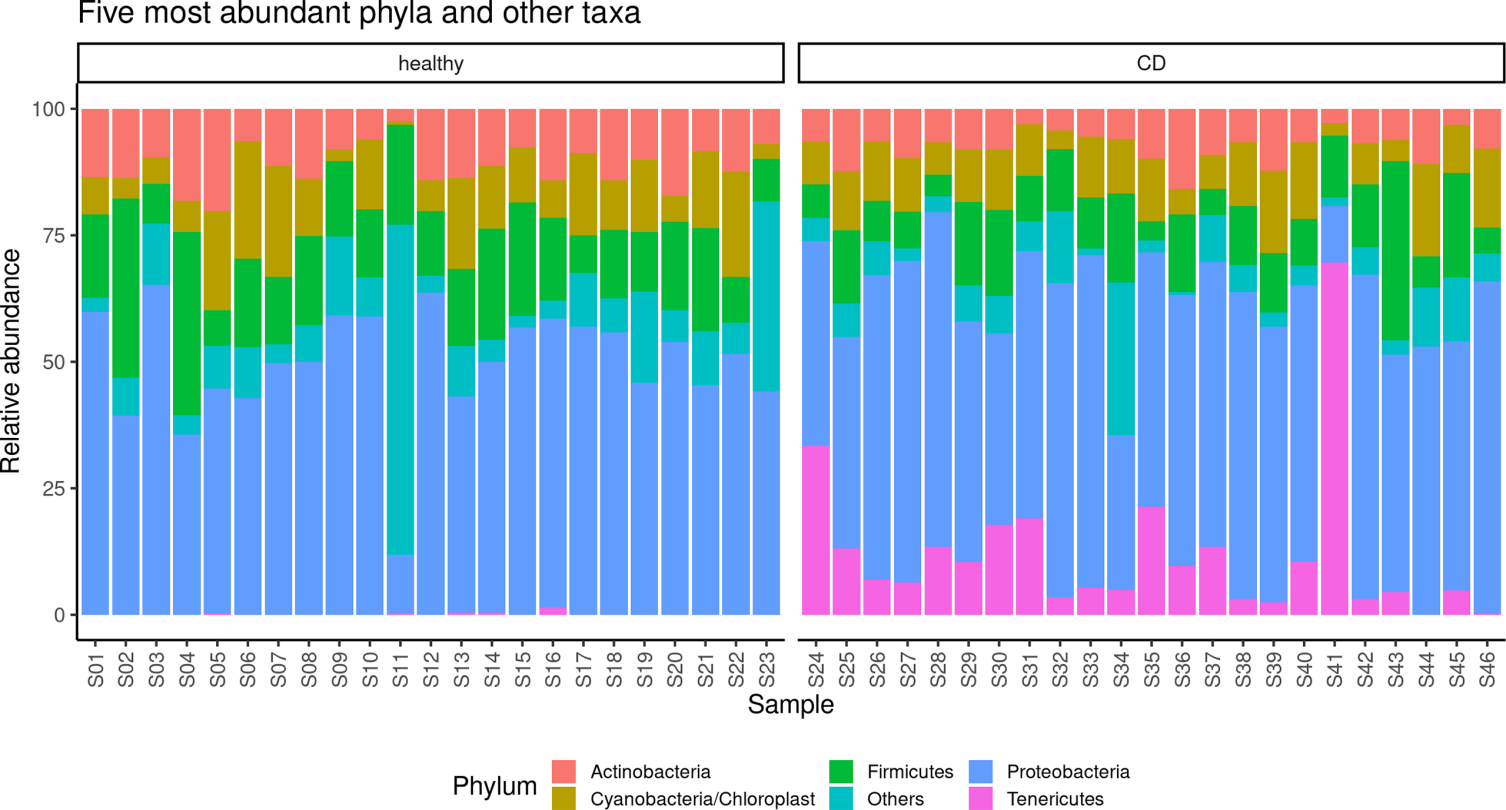
Taxonomic composition of the paraffin–embedded ileal samples in ileal and CD tissues. The stacked bar plot shows the relative abundance of the five more abundant bacterial phyla in each sample, the “Others” group contains phyla with ranks below five.

The alpha diversity of the samples displayed significant differences between CD and healthy tissues for the Chao (*p* = 0.014) and Shannon indexes (*p* = 0.045) (**Supplementary Figure 1**), while no differences were observed for the evenness index. The differences in the Chao index may evidence that rare OTUs are enriched in healthy *vs*. CD tissues. The analysis of the taxonomic composition revealed that more than 89% of the sequences collected were classified into five phyla: *Proteobacteria* (50.6%), *Firmicutes* (14.1%), *Actinobacteria* (9.5%), *Cyanobacteria* (10.6%), and T*enericutes* (6.1%). The relative abundance of the five most represented microbial phyla in CD and healthy tissues is reported in **Figure 2**.

In detail, in the affected tissue, we observed a particular enrichment of the *Tenericutes* phylum which is relatively absent in healthy tissue (**Figure 2**). In addition, we also explored any genus differences between the two considered settings, and the relative abundance of the five most represented microbial genera in CD and healthy tissues is reported in **Supplementary Figure 2**. Notably, we observed a particular enrichment of the *Mesoplasma* genus (belonging to the *Tenericutes* phylum) which is relatively absent in non–inflamed tissue.

**Figure 3:**
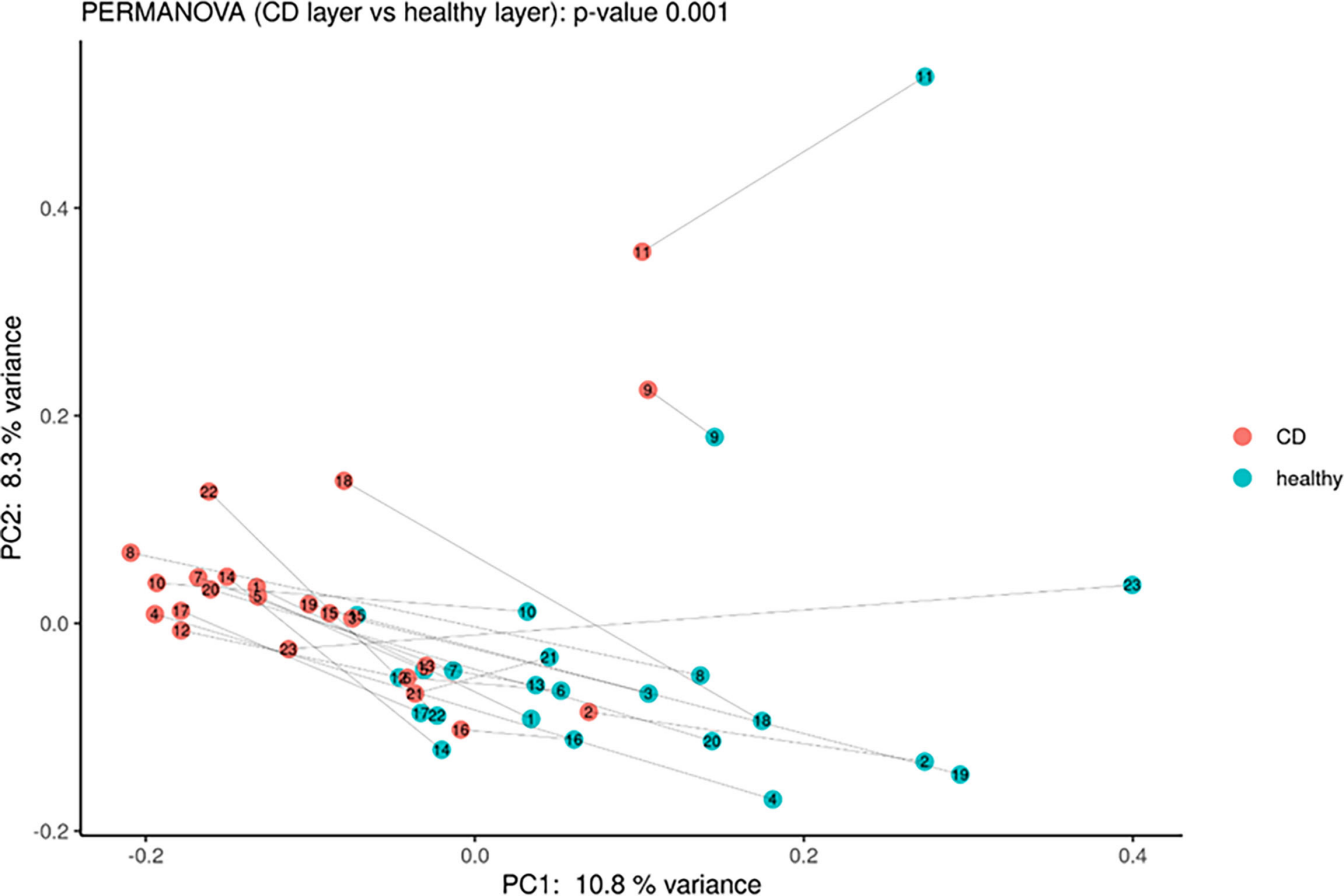
Principal coordinate analysis using Bray–Curtis dissimilarity as a distance metric on square root–transformed percent abundance of identified OTUs showing permuted *p*–value of general β dispersion and pairwise β dispersion of CD and healthy groups. The lines connect the samples from the same patient.

Moreover, PCoA analysis revealed significant differences between healthy *vs*. inflamed tissues (**Figure 3**), and it was possible to detect a clear clustering between the two groups. Finally, the paired comparison of the abundance of single OTUs revealed significant [*p* < 0.05, abs (logFC) ≥ 1] differences between the two sample groups.

**Figure 4:**
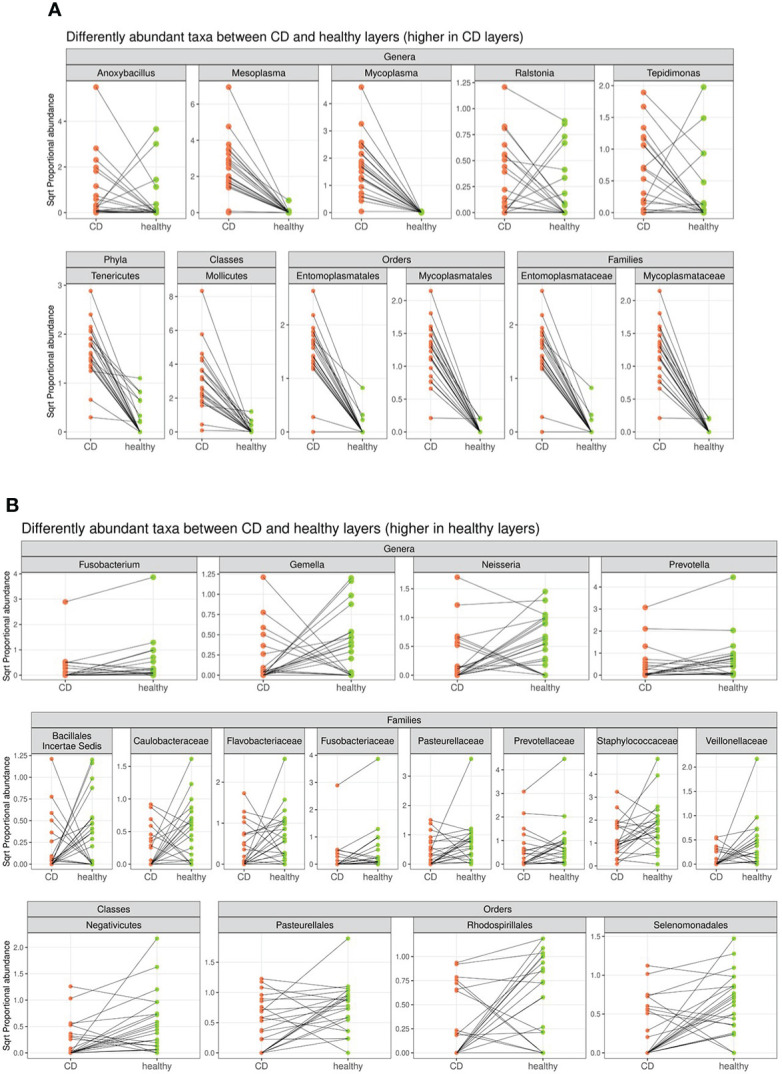
Segment plot depicting significantly different taxa (phylum, classes, families, orders, and genera) between CD **(A)** and healthy tissues **(B)**. Lines connect paired samples and highlight the differences in normalized abundance for the indicated rank. Red or blue colors highlight a decrease or increase in CD *vs*. healthy tissues, respectively. Numbers on the top–left corner represent counts of decreased (red) and increased (blue) measurement for paired samples. Plot titles report the shrunken log2 fold change between inflamed and non–inflamed tissues (according to the DESeq2 function lfcShrink). All taxa tested have a *p*–value <0.05.

In detail, the phylum *Tenericutes*, the class *Mollicutes*, the orders *Entomoplasmatales* and *Mycoplasmatales*, the families *Entomoplasmataceae* and *Mycoplasmataceae*, and the genera *Mesoplasma*, *Mycoplasma*, *Anoxibacillus*, *Ralstonia*, and *Tepidimonas* were significantly higher in CD tissue (**Figure 4**). On the contrary, the class *Negativicutes*, the orders *Pasteurellale*s, *Rodospirillales*, and *Selenomonadals*, the families *Prevotellaceae*, *Flavobacteriaceae*, *Fusobacteriaceae*, *Pasteurellaceae*, *Caulobacteriaceae*, *Veillonellaceae*, and *Staphylococcaceae*, and the genera *Fusobacterium*, *Prevotella*, *Neisseria*, and *Gemella* were significantly lower in CD tissue (**Figure 4**). This result indicates a different regulation of the mucosal microbial population in the two different settings, possibly due to the different inflammatory environment that guides the selection of certain bacterial populations over others. The graphical results of the differential analysis of all taxonomic ranks are available in **Figures 4B**.

### Differences between first surgery *vs*. surgical relapse

#### Dissimilar composition of the ileal microbiota

Based on clinical characteristics, we divided the patients into two groups (a: first surgery and b: relapse) and compared the respective microbial patterns in CD and healthy tissues. Starting from the different comparisons, we have documented some significant variations.

**Figure 5:**
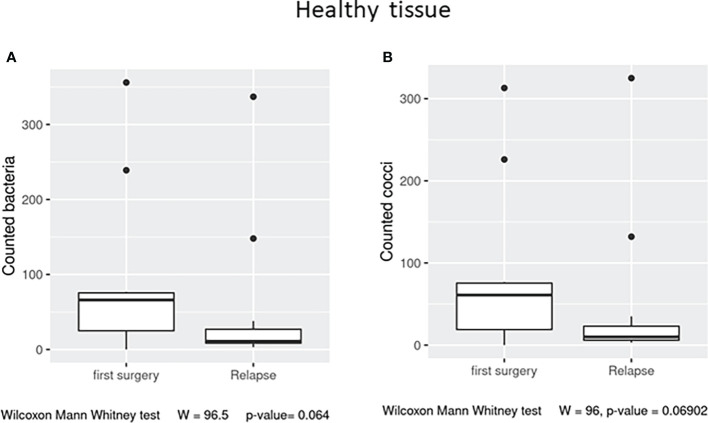
Box plot distribution of adherent bacteria counts **(A)** and cocci **(B)** identified in healthy samples from the first surgery and relapse patients. Analyses were assessed using the Mann–Whitney paired test, and *p*–values less than 0.05 were considered statistically significant.

In the healthy tissue, we observed a trend (*p* = 0.06) in the number of observed total bacteria and cocci, enriched in the first surgery patients (**Figures 5A, B** **)**, while in the affected tissue, no significant differences were detected (data not shown).

The alpha diversity of the samples displayed no significant differences for Chao, Shannon, and evenness indexes (**Supplementary Figures 3A)** in CD and healthy tissues for both patient groups (**Supplementary Figures 3A)**. In addition, beta diversity displayed no significant clustering in CD and healthy tissues for both subsets of patients (**Supplementary Figures 4A)**.

**Figure 6:**
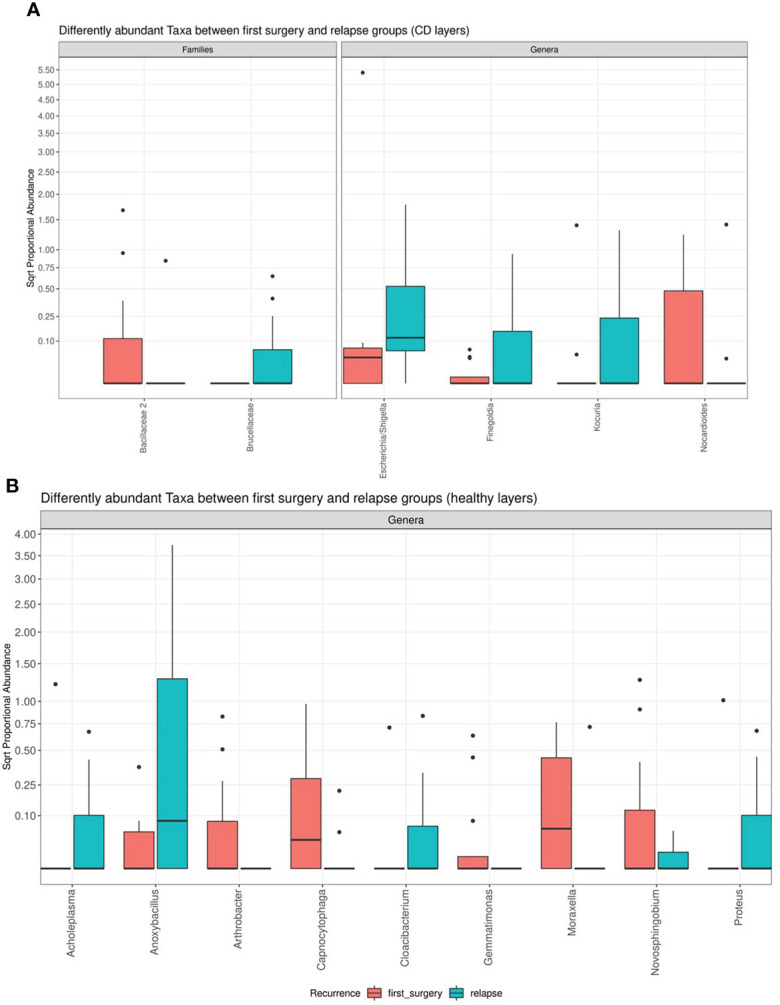
Box plot showing the results of taxa differential abundance analysis between the first surgery and relapse patients, respectively, in CD **(A)** and healthy tissues **(B)**. The *y*–axis has been scaled to improve the readability of values. All results have an adjusted *p*–value <0.05.

However, the differential abundance analysis revealed significant differences. In detail, regarding the CD tissue, the family *Bacillaceae 2* and the genus *Nocardioides* were significantly higher in the first surgery compared to the relapse patients, on the contrary, the family *Brucellaceae* and the genera *Escherichia/Shigella*, *Finegoldia*, and *Kocura* were significantly enriched in the relapse condition (**Figure 6**).

The analysis of the healthy tissue revealed that all differences were focused on several genera: in particular, *Antrobacter*, *Capnocytophaga*, *Gemmatimonas*, *Moraxella*, and *Novosphingobium* were all significantly augmented in the first surgery patients, while *Acholeplasma*, *Anoxibacillus*, *Cloacibacterium*, and *Proteus* were decreased (**Figure 6** **)**. The meaning of these data may be linked to a perturbation of ileal microbiota architecture at surgery time, with a consequent different microbiota recolonization after ICR.

#### Discrepancies in the plasma levels of miR–155, miR–223, and miR–423

**Figure 7:**
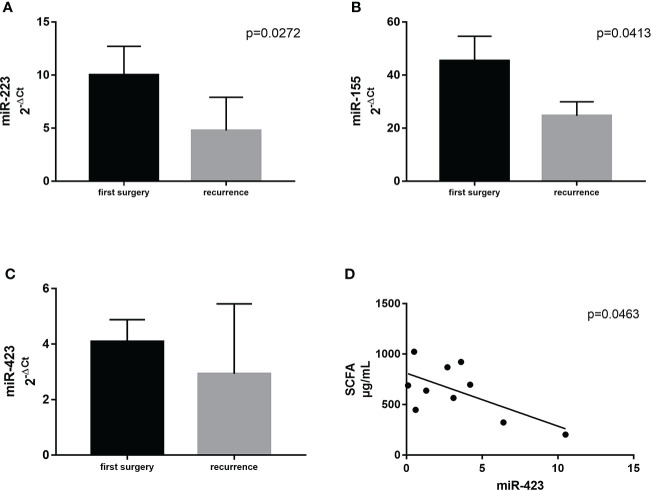
Expression levels of miR–223 **(A)** and miR–155 **(B)** in plasma samples from CD patients with surgical recurrence (*n* = 12) compared to those at first surgery (*n* = 16). Correlation between plasma levels of miR–423 and the total amount of SCFA in the serum of the same patient **(C)**. **(D)** Correlation between SCFA and miR–423. Data are expressed as mean ± SD.

Supposing that significant variations in ileal microbial architecture could be mirrored in circulating factors, associated with GM composition and expression, we selected three specific circulating miRNAs related to GM activity and the regulation of inflammation (37). In detail, we measured miR–155, miR–223, and miR–423. We observed significant differences between the first surgery and relapse patients in terms of miR–223 (*p* = 0.0273) and miR–155 (*p* = 0.0413), both being downregulated in recurrence patients compared to patients at first surgery (**Figures 7A** **)**. miR–423 exhibited a similar trend without reaching statistical significance (**Figure 7**). This result suggested the activation of different signaling pathways in relapse patients compared to those at first surgery. In addition, this outcome may also indicate a dissimilar regulation of the activated inflammatory pathways involved in CD recurrence compared to first surgery.

#### Different serum FFA profiles

**Figure 8:**
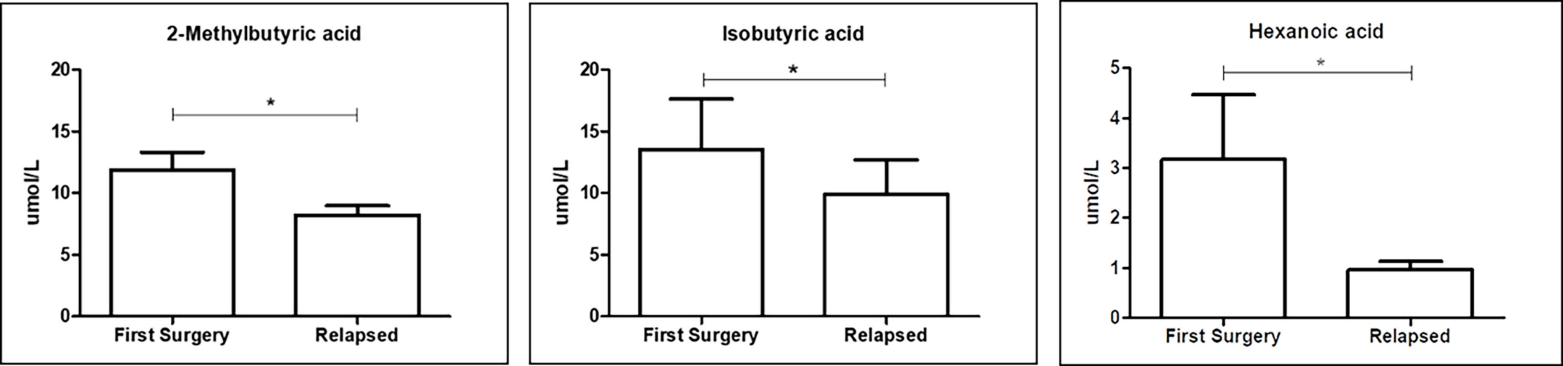
Box plot reporting the statistically significantly different FFAs between the first surgery and relapse patients. Analyses were assessed using the Mann–Whitney test, and *p*–values less than 0.05 were considered statistically significant. The asterisks (*) represent p–values: *p < 0.05.

The production of SCFAs, the major bacterial fermentative end–products, reflects the intestinal microbiota composition and especially its function. In addition, given that SCFAs are crucial to health, maintaining the host gut physiology, and that a part of them enters the systemic circulation (15), it is tempting to speculate that GM composition could be also related to FFA levels. We then performed qualitative and quantitative analyses of serum FFAs, namely, linear SCFAs (acetic, propionic, butyric, and valeric acids), branched SCFAs (isobutyric, isovaleric, 2–ethylhexanoic, 2–methylbutyric, and cyclohexanoic acids), MCFAs (hexanoic, heptanoic, octanoic, nonanoic, decanoic, and dodecanoic acids), and LCFAs (tetradecanoic, hexadecenoic, and octadecanoic acids), between the first surgery and relapse patients. We observed significantly higher levels of two branched SCFAs, notably 2–methyl butyric (*p* = 0.0380) and isobutyric acids (*p* = 0.0245), and one MCFA, namely, hexanoic acid (*p* = 0.0492), in relapse patients (**Figure 8**). The meaning of this result may be connected to a different expression of GM functionality between the first surgery and relapse patients.

#### Correlation between miRNA profile, FFAs, and ileal microbiota

Firstly, we correlated the levels of miR–155, miR–223, and miR–423 with FFA profiles. Among all the correlations, we identified only a negative association between miR–423 and the total amount of SCFA in the serum (*p* = 0.0463) (**Figure 7**).

Next, in order to assess particular associations between bacterial composition in ileal tissue and circulating bacterial metabolites, as well as miRNAs, we correlated the levels of the two SCFAs that were significantly changed between the first surgery and relapse patients (2–methyl butyric and isobutyric acids) with clades significantly altered in the same conditions (both CD and healthy settings). In addition, we also assessed the associations with hexanoic acid.

**Figure 9:**
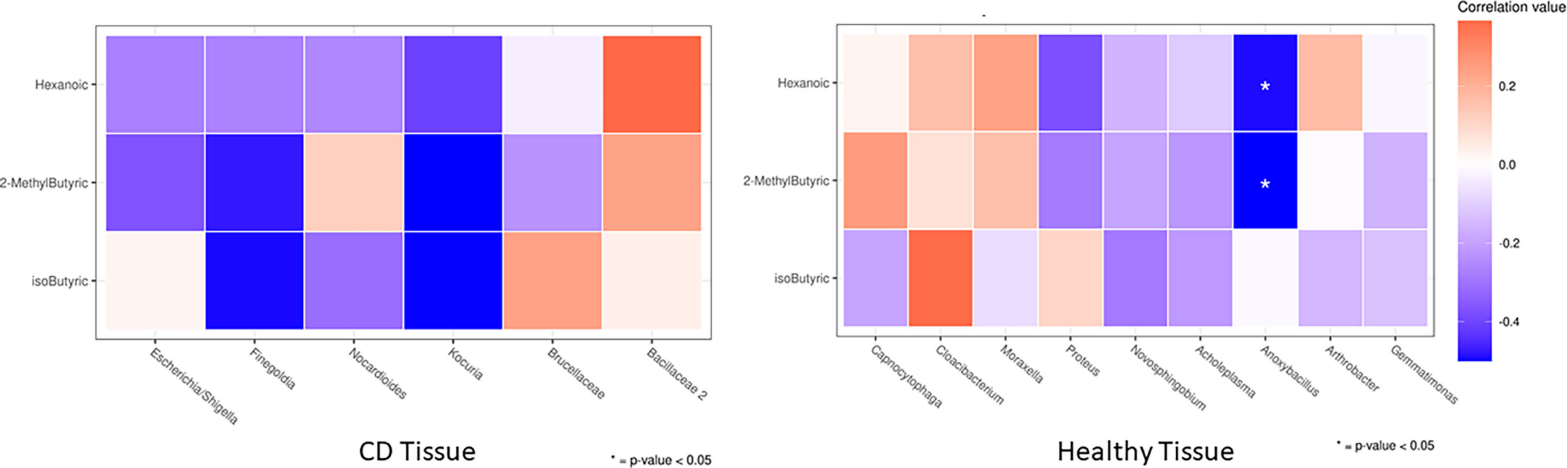
Heatmap of the Spearman correlation values between significantly changed FFAs (rows) and taxa that were differently abundant in DESeq2 analyses (columns) between the first surgery and relapse patients. As it is an explorative analysis, non–adjusted *p*–values lower than 0.05 are marked with an asterisk.

We documented the significant association only in the healthy setting, notably, the genus *Anoxybacillus* (greatly enriched in the healthy ileal tissue of relapse patients) was negatively correlated (*p* < 0.05) with circulating 2–methyl butyric and hexanoic acids (both reduced in the serum of relapse patients). The correlation heatmap in CD and healthy tissues is shown in **Figure 9**, and it is relevant because it compares the obtained Spearman correlations in the two analyzed settings, thus suggesting a potential involvement of healthy tissue microbiota in the regulation of circulating serum inflammatory markers.

In the same way, we correlated the significantly changed miRNAs between the first surgery and relapse patients (miR–223 and miR–155) with the abovementioned selected genera. However, any significant correlation was found (data not shown).

### Risk factors for recurrence

By logistic regression analysis, among all the considered parameters, we identified only the serum hexanoic acid as the significant independent risk factor for surgical recurrence: patients with hexanoic acid <1.4 µM (median value) were at increased risk of recurrence compared to patients exhibiting higher serum level of this MCFA (OR 18, 95% confidence interval 1.24–261.81, *p* = 0.006). If confirmed by future validated data, this outcome suggests that monitoring of hexanoic acid level might be useful as a potential strategy for recurrence predictor.

## Discussion

A deeper understanding of CD recurrence mechanisms may help clinicians refine treatment and improve outcomes. Recent evidence supports the key role of an aberrant immune response to the microbiota in the development of gut mucosal inflammation, in both CD pathogenesis and recurrence. However, the mechanisms regulating the interplay between the host immune system and ileal microbiota, as well as the specific changes occurring in GM composition and functionality, remain to be defined. To shed light on these intricate relationships between the actors of the “microbiota–immunity” axis, in our previous pilot study, we explored this connection at the tissue level, analyzing the molecular immune response distribution within the ileal layers and evaluating the correlated mucosal microbiota in pathological/healthy settings and in the first surgery/relapse clinical conditions (14). Having observed a dissimilarity of ileal cytokine distribution and mucosal microbiota composition in the first surgery and relapse patients, we wondered if even at the systemic level the microbiota–immunity axis could be differently regulated, leading to new possible recurrence circulating biomarkers’ detection (such as miRNAs and FFAs).

To this aim, in this study, we explored the microbiome–immunity axis at the systemic level, in a larger number of patients (10 of the 28 patients were shared between the two studies). In detail, we assessed ileal microbiota composition, systemic functionality, and immunoregulation at the gene expression level in the first surgery and recurrence conditions and at the two different inflammatory tissue settings.

Firstly, the GM composition of CD patients was evaluated in paraffin–embedded materials. Several findings suggest that GM changes in CD, including decreased microbial diversity and relative abundance, particularly of bacteria (38). CD clinical lesions are usually seen in the distal ileum and colon, both of which have significant microbial concentrations. We investigated the bacterial wall adherence in healthy and CD ileal tissues, in line with previous results (38), we reported enrichment of total bacteria count in healthy tissue, with an increase in bacillus morphology. On the other hand, the pathological tissue appeared depleted of bacteria, suggesting a dysbiotic condition. Indeed, CD patients usually present dysbiosis and reduced biodiversity in the GM composition, linked to an increase in pro–inflammatory bacteria and a decrease in anti–inflammatory bacteria (39). So, in order to explore the taxonomy and, consequently, the inflammatory nature of adherent GM, we performed an in–depth taxonomic analysis. According to our previous pilot results (14), the majority of the sequences collected in both tissues (affected and healthy) were classified into five phyla: *Proteobacteria* (50.6%), *Firmicutes* (14.1%), *Actinobacteria* (9.5%), *Cyanobacteria* (10.6%), and T*enericutes* (6.1%). In general, CD patients usually show a decreased number of *Firmicutes* (40), on the other hand, the *Proteobacteria* phylum has a well–preserved ecological pattern associated with inflammation (41). Nevertheless, we are aware that the quantity of *Pseudomonas*, a typical paraffin–embedded contaminant genus, may have an effect on the amounts of *Proteobacteria* detected (42), as we also reported in our previous study (14).

Successively, we compared the bacterial architecture between the two examined settings (CD *vs*. healthy), finding significant differences in alpha diversity expressed through the Chao and Shannon indexes. Confirming our previous pilot outcomes (14), the principal coordinate analysis documented different microbial distribution between the two evaluated tissues. In detail, we verified an increase of the *Tenericutes* phylum, comprising *Mycoplasma*, in CD tissue. From a biological point of view, *Mycoplasma* has a role in promoting inflammation, indeed, lipopeptides from mycoplasmal membranes have been postulated to promote chronicity and higher immune responses than other bacteria [as reviewed in (43)]. Additionally, the *Prevotella* genus was lower in CD tissue. *Prevotella* prevalence is related to increased Th17–mediated mucosal inflammation, which is consistent with the capacity shown by *Prevotella* to drive Th17 immunological responses *in vitro.* In line with our result, a study indicated reduced *Prevotella* in pediatric Crohn’s disease (44). Furthermore, the most comprehensive study to date found no association between *Prevotella* and new‐onset Crohn’s disease before treatment (45).

Previous studies documented that distinct profiles of mucosa–associated microbiota at the ICR and postoperatively are associated with disease recurrence and remission, respectively (46). On these premises, as well as based on our previous pilot study (14), we performed a differential abundance analysis between the relapse and first surgery patients. We did not observe significant alterations between the two conditions at the microscopy levels for both CD and healthy settings, nevertheless, significant differences in microbiota signatures, mostly at the genus level, were detected with NGS, pointing to a different microbiota recolonization after ICR, as evidenced by a recent study (47). However, compared to our pilot study, due to the higher number of samples, we were able to further find different taxa (**Figures 6A** **)**. Indeed, regarding the affected tissue, the family *Bacillaceae* was significantly higher in the first surgery patients compared to the relapse patients. An increase in *Bacillaceae* was recently observed in UC patients, in relation to healthy controls (48). This family is composed of aerobic spore–formers, and their presence in the gut is related to the ingestion of food and water. *Bacillaceae* is one of the rare *Firmicutes* families that share a specific LPS, being highly immunogenic. Indeed, it has recently been suggested that the efficiency of the immune system may depend on the immunogenicity of microbiota lipopolysaccharides (49). On the contrary, the family *Brucellaceae* and the genera *Escherichia/Shigella*, *Finegoldia*, and *Kocura* were significantly increased in the relapse condition. *Brucellaceae* is a family of Gram–negative bacteria involved in the progression of diseases of the central nervous system, including those of affective and psychiatric nature (50). Moreover, *Escherichia/Shigella* is a pro–inflammatory genus related to intestinal inflamed conditions, as it was previously found enriched in CRC patients (30, 51).

In healthy tissue, we observed that *Antrobacter*, *Capnocytophaga*, *Gemmatimonas*, *Moraxella*, and *Novosphingobium* were all significantly augmented in the first surgery patients, while *Acholeplasma*, *Anoxibacillus*, *Cloacibacterium*, and *Proteus* were decreased. In particular, *Proteus* belonging to Gram–negative facultative anaerobic bacilli has been recently linked to CD recurrence after surgery (52). We hypothesized an ICR influence on microbiota architecture based on our observations of substantial variations between the first surgery and recurrence patients, possibly due to the removal of the ileocecal valve (ICV) as discussed in our previous work (14).

Bacterial dysbiosis in CD patients increases intestinal barrier permeability, which explains the pathophysiology of luminal translocation of bacteria and their products (SCFAs) at the systemic level, a common occurrence in CD, which promotes a persistent inflammatory response in these patients. Many recent studies have suggested that bacterial product translocation causes uncontrolled systemic inflammation in CD patients, due to the host’s circulating epigenetic fragments (miRNAs), which are gut bacteria–associated. In detail, the circulating miRNAs are attractive non–invasive biomarkers with promising clinical values (53). However, few studies have been performed on the relationship between miRNAs and the different clinical CD conditions.

While in our previous pilot study we focused only on the interplay of microbiota and immune response at the tissue level, here, we studied also some microbial aspects of systemic inflammation. miR–155 is a multifunctional miRNA, involved in several biological processes such as hematopoiesis, inflammation, and immunity, its putative targets include several molecules of NF–κB signaling and the suppressor of cytokine signaling (SCS1). miR–155 is reported to be upregulated in the inflamed colonic mucosa of patients with active UC (54–56). However, it plays both positive and negative regulatory roles in immune responses, and it was hypothesized that miR–155 is part of a negative feedback loop to dampen inflammatory responses (57). Here, we reported a significant decrease in the level of miR–155 in the plasma of patients with surgical recurrence compared to those at the first surgery, suggesting the activation of different signaling pathways among these patients that deserve to be explored, to identify the molecular mechanisms underlying the recurrent relapses observed only in a subgroup of CD patients. We also observed the same significant decrease for miR–223, a potent regulator of some inflammatory responses. Its altered expression has been linked to several immune disorders, including rheumatoid arthritis and type 2 diabetes mellitus. It is differentially expressed during macrophage polarization, and miR–223–deficient macrophages were hypersensitive to LPS stimulation and exhibited delayed responses to IL–4 (58).

Regarding miR–423, we did not observe a significant difference between the first surgery and recurrence patients, however, we detected a negative correlation with hexanoic acid. In other words, when the hexanoic level is low (a risk factor for recurrence), miR–423 increases. This is an intriguing result as in a recent work, miR–423, inserted in a specific miRNA signature, has shown a strong capacity to confirm the presence of recurrence within 1 year of surgery, which could also help spare patients from colonoscopies (59). It has been observed that this miRNA appears with the establishment of recurrence and could therefore be participating in the development of active lesions in CD (59).

In addition, it has been reported in the literature that there were differences in the GM and in microbial metabolism depending on CD disease activity (60). We observed a significant decrease of two circulating branched–chain fatty acids (BCFAs), 2–methylbutyric and isobutyric acids, in recurrence conditions. It is known that BCFAs are mainly produced during the fermentation of branched–chain amino acids, such as valine, leucine, and isoleucine, by the GM (61). In the human intestine, the fermentation of BCFAs is carried out mainly by the genera *Bacteroides* and *Clostridium* (62). Moreover, in our study, recurrence patients exhibited diminished levels of hexanoic (caproic) acid, an MCFA whose metabolic fate is β–oxidation to acetyl–CoA, oxidized in the tricarboxylic acid cycle or converted to ketone bodies. Hexanoic acid reduces the colonization and dysbiotic expansion of potentially pathogenic bacteria in the gut (63). Even more recently, MCFAs have been shown to support the differentiation of some T helper (Th) lymphocytes, specifically Th1 and Th17, and to suppress the development of regulatory T cells (Tregs) (64). Hexanoic acid, in particular, seems to be prototypically endowed with such pro–inflammatory properties, which result from the activation of p38 MAPK signaling (64). Notably, it can also be directly produced by some of the microbiota bacteria such as *Prevotella*. In line with our result, fecal levels of hexanoic acid were recently shown to be inversely correlated with CD activity (65). In addition, our logistic regression analysis identified serum hexanoic acid as a significant independent risk factor for surgical recurrence. However, we are aware of the limitation of this result, and to translate these functional data into a more profound biological disease understanding, more knowledge of the relevance of this metabolite modulation is needed.

So far, FFA profiling mainly detects associations between profiles and specific phenotypes, which may not always be meaningful. In addition, we have to explore further whether changes in metabolites are the cause or consequence of particular CD conditions. Integration of other “omics” may enable a further understanding of IBD–related pathophysiological processes.

Finally, in order to explore a possible different regulation of GM functionality at the first surgery and recurrence conditions, we evaluated the potential associations between significantly changed clades and FFAs in CD vs. healthy settings in two clinical conditions (**Figure 9**).

We observed a particular negative association of *Anoxybacillus* (a bacillus significantly increased in healthy tissue of recurrence patients) with 2–methyl butyric and hexanoic acids, both reduced in the serum of recurrence patients. The genus *Anoxybacillus* is composed of 22 species, and it is interesting to highlight that some of them showed immunomodulatory and immunostimulatory properties due to the production of specific metabolites (66, 67). Our outcome may suggest a direct association of this microbial pattern in CD recurrence, however, we are not yet able to give mechanistic explanations, which require a study on cellular or *in–vivo* models.

Interestingly, in a previous study on CRC, according to the bacterial driver–passenger model put forward by Tjalsma et al. (68), *Anoxybacillus* was included in the group of driver bacteria for CRC, being away from the tumor sites (i.e., adjacent non–malignant tissue) (69). This model states that CRC development is triggered by local mucosal colonization of drivers that cause changes in the tumor microenvironment allowing for colonization by opportunistic (passenger) bacteria or their by–products, i.e., metabolites, to pass through the epithelium, easing disease progression (70). Each bacterium contributes to carcinogenesis by a distinct microbial signature, such as the production of deleterious metabolites or by–products, stimulation or inhibition of local immune responses, or modulation in gene expression. Making a parallelism with the CRC bacterial driver–passenger model, we may speculate that the microbial patterns that lead to the onset of recurrent inflammation could be triggered by particular “driver” bacteria that reside in healthy tissue. They may activate an aberrant local immune response, leading to an increase in gut permeability and translocation of microbial products in the systemic circulation, promoting a vicious cycle with the production of epigenetic regulators at the systemic level acting as inflammation amplifiers. To corroborate this hypothesis, it is important to notice that there is a recognized increased risk for CRC in patients with IBD due to the extent of inflammatory change.

Even so, due to the small number of samples and the use of paraffin–embedded methods (which are prone to biases introduced by the embedding process and tissue archiving time), such analyses should be improved in future studies to adequately describe the complex scenario of the relationship between circulating pro– and anti–inflammatory factors and intestinal microbiota composition. Nonetheless, we are conscious that more human and animal model studies will be required to reinforce our results, but there is no dispute about the study’s innovativeness.

## Conclusion

To sum up, this study was proposed as a continuation and expansion of our previous pilot study (14) in which we examined the relationships between immune response elements (represented by cytokine levels in the serosa, submucosa, and mucosa) and microbiota at the tissue level, in the first surgery and relapse CD patients, demonstrating a different regulation among the examined clinical conditions. We therefore studied the architecture of mucosal GM in the same conditions as the pilot study (14) but in a greater number of patients, confirming a different ileal microbiota composition and abundance.

Unlike our previous study, in this research, we explored further the differences at the systemic level, observing for the first time a dissimilar regulation of ileal microbiota and circulating microbial–associated inflammatory factors (SCAFs, BCFAs, and miRNAs) between the first surgery and relapse CD patients, suggesting a different involvement of the gut microbiota–immunity axis in the two clinical conditions. Moreover, we also detected an association between the serum hexanoic acid and recurrence risk that needs to be further explored with prospective studies. Indeed, a comprehensive and accurate analysis is imperative to identify biomarkers of recurrence, performing targeted therapies in CD. Finally, we observed for the first time on healthy ileal tissue the potential involvement of the microbiome pathways in triggering recurrence determinants, pointing out that also GM eubiosis maintenance may be crucial for CD early treatment adjustments. Nonetheless, further mechanistic studies evaluating cellular and humoral immune responses, GM alterations, and epigenetic predisposition will help clinicians to better control and personalize the management of patients with CD, thus avoiding future post–surgery recurrences.

## Data availability statement

The microbial–related data (raw reads, OTU tables, and taxonomic assignments) are freely available at NCBI Gene Expression Omnibus under the series accession GSE162844 and GSE198329, and the analysis scripts (in 263 R) are available at GitHub (https://github.com/matteoramazzotti/papers/tree/main/2022IBD).

## Ethics statement

The studies involving human participants were reviewed and approved by Careggi University Hospital. The patients/participants provided their written informed consent to participate in this study. Written informed consent was obtained from the individual(s) for the publication of any potentially identifiable images or data included in this article.

## Author contributions

ER, AA, and FG designed the study. ER, MD, and CL revised the literature on this topic. MD, ER, SB, LaC, GN, and Arcese D.A. collected the samples. LoC, MD, ER, SB, MP, and EB performed the experiments. ER, CL, LoC, MD, EB, and SB analyzed the data. LG and MR performed the microbiota analysis. ER wrote the manuscript. ER edited the manuscript. ER, FG, and AA supervised the preparation of the manuscript. AA, FG, ER, SS, GB, CM, and CL revised the manuscript. FG, ER, CL, and AA were responsible for funding acquisition. All authors contributed to the article and approved the submitted version.

## Funding

This work was supported by Fondazione Cassa di Risparmio di Firenze (CRF2018, Number 2016.0842) and was funded by ECCO Grant 2020 to FG. Moreover, this project received funding from MUR under the umbrella of the European Joint Program Initiative “A Healthy Diet for a Healthy Life” (JPI–HDHL) and of the ERA–NET Cofund ERA–HDHL, ID: 1523 (GA N 696295 of the EU HORIZON 2020 Research and Innovation Programme).

## Acknowledgments

We thank Professor Marcus J. Claesson for critically reading the manuscript. We also sincerely thank the patients for their willingness to participate in the study.

## Conflict of interest

The authors declare that the research was conducted in the absence of any commercial or financial relationships that could be construed as a potential conflict of interest.

## Publisher’s note

All claims expressed in this article are solely those of the authors and do not necessarily represent those of their affiliated organizations, or those of the publisher, the editors and the reviewers. Any product that may be evaluated in this article, or claim that may be made by its manufacturer, is not guaranteed or endorsed by the publisher.

## Supplementary material

Supplementary Figure 1 Box-plots showing alpha diversity indices (Chao1 index, Shannon index, Evenness) in CD and healthy samples. Statistical differences were evaluated using paired Wilcoxon signed-rank test for Chao, Shannon and Evenness indices. P-values less than 0.05 were considered statistically significant.

Supplementary Figure 2 Taxonomic composition of paraffin-embedded ileal samples in ileal and CD tissue. The stacked bar plot shows the relative abundance of the five more abundant bacterial genus in each sample, The “Others” group contains genus with ranks below five.

Supplementary Figure 3 Box-plots reporting alpha diversity indices (Chao1, Shannon and Eveness index) in first surgery and relapse patients, for both CD **(A)** and Healthy tissues **(B)**. P-values less than 0.05 were considered statistically significant.

Supplementary Figure 4 Principal Coordinate Analysis using Bray-Curtis dissimilarity as a distance metric on square root transformed percent abundance of identified OTUs showing permuted p-value of general β dispersion and pairwise β dispersion of CD and healthy groups. The lines connect the samples from the same patient.
