# Supplementary figures and images for: Crohn’s disease recurrence updates: first surgery vs. surgical relapse patients display different profiles of ileal microbiota and systemic microbial-associated inflammatory factors
Source: Front Immunol. 2022 Jul 29;13:886468. doi: 10.3389/fimmu.2022.886468 (PMC9374303; doi:10.3389/fimmu.2022.886468)

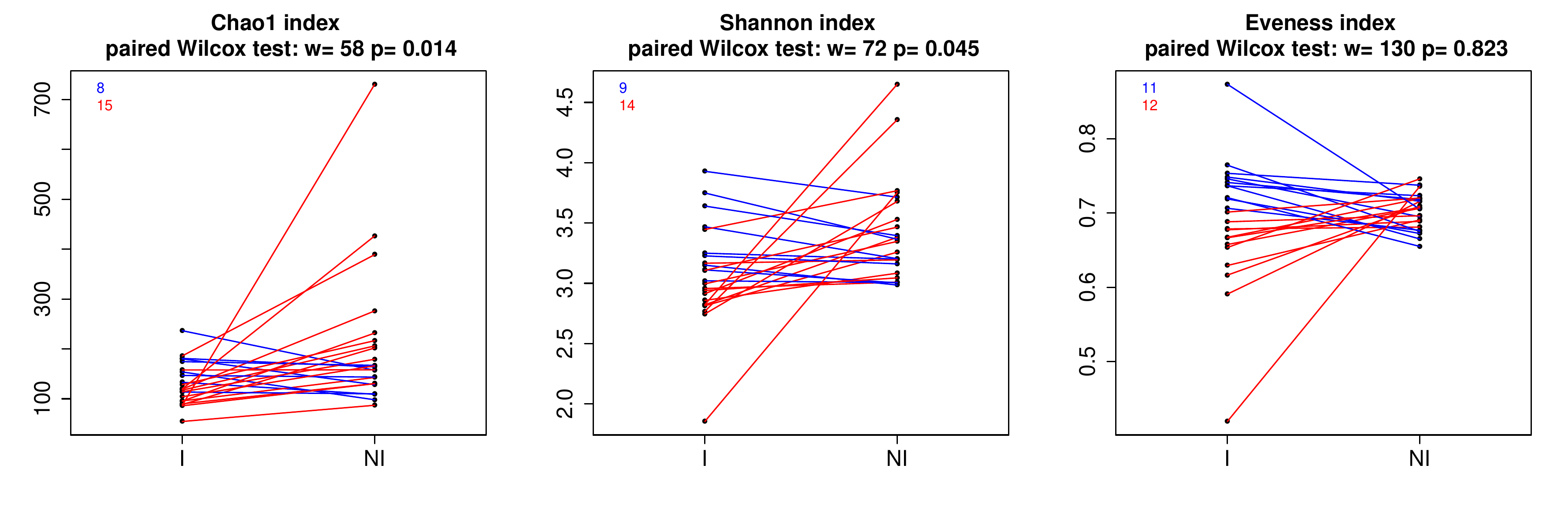

Supplement: Supplementary Figure 1 — Box-plots showing alpha diversity indices (Chao1 index, Shannon index, Evenness) in CD and healthy samples. Statistical differences were evaluated using paired Wilcoxon signed-rank test for Chao, Shannon and Evenness indices. P-values less than 0.05 were considered statistically significant. [file Image_1.tif]

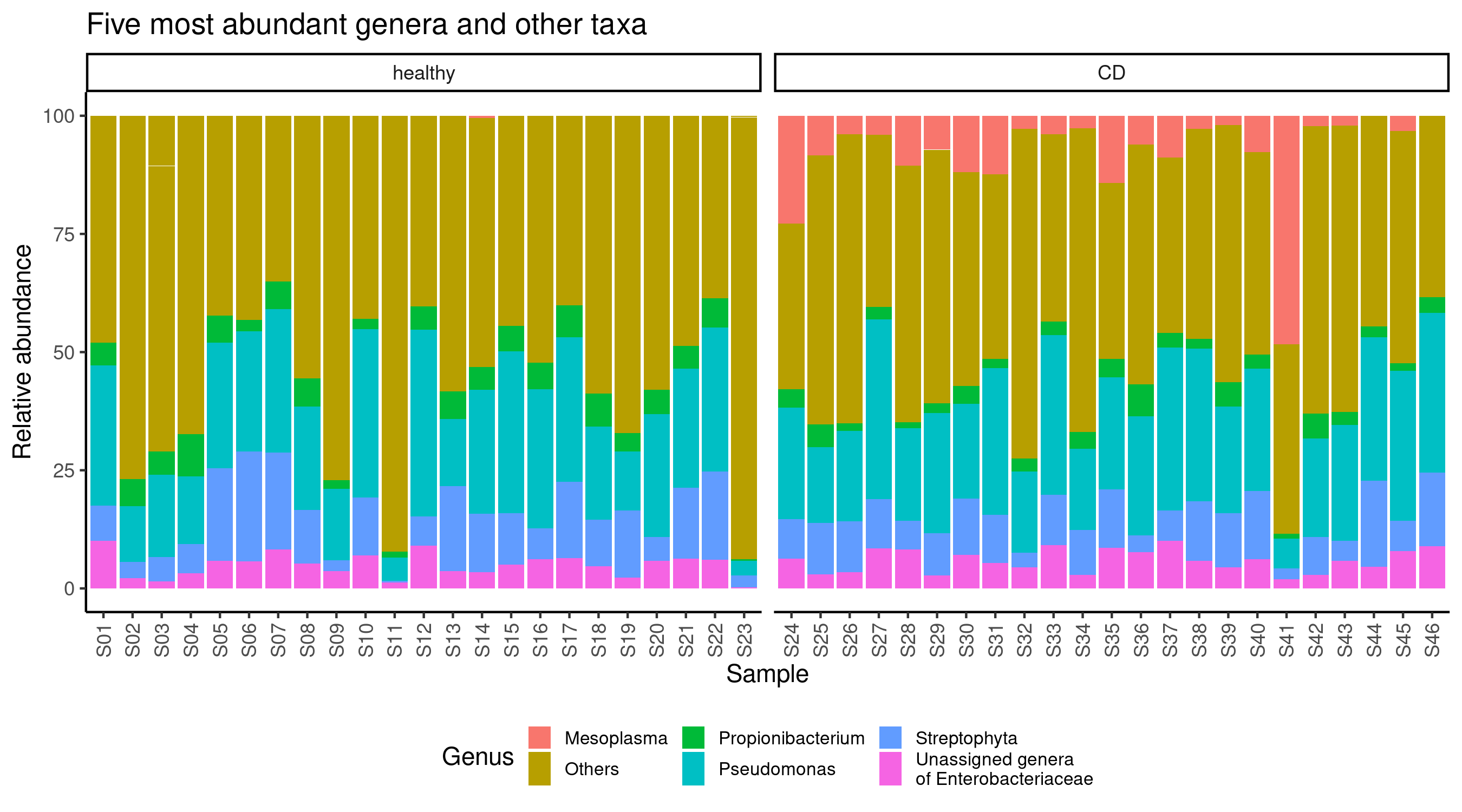

Supplement: Supplementary Figure 2 — Taxonomic composition of paraffin-embedded ileal samples in ileal and CD tissue. The stacked bar plot shows the relative abundance of the five more abundant bacterial genus in each sample, The “Others” group contains genus with ranks below five. [file Image_2.tiff]

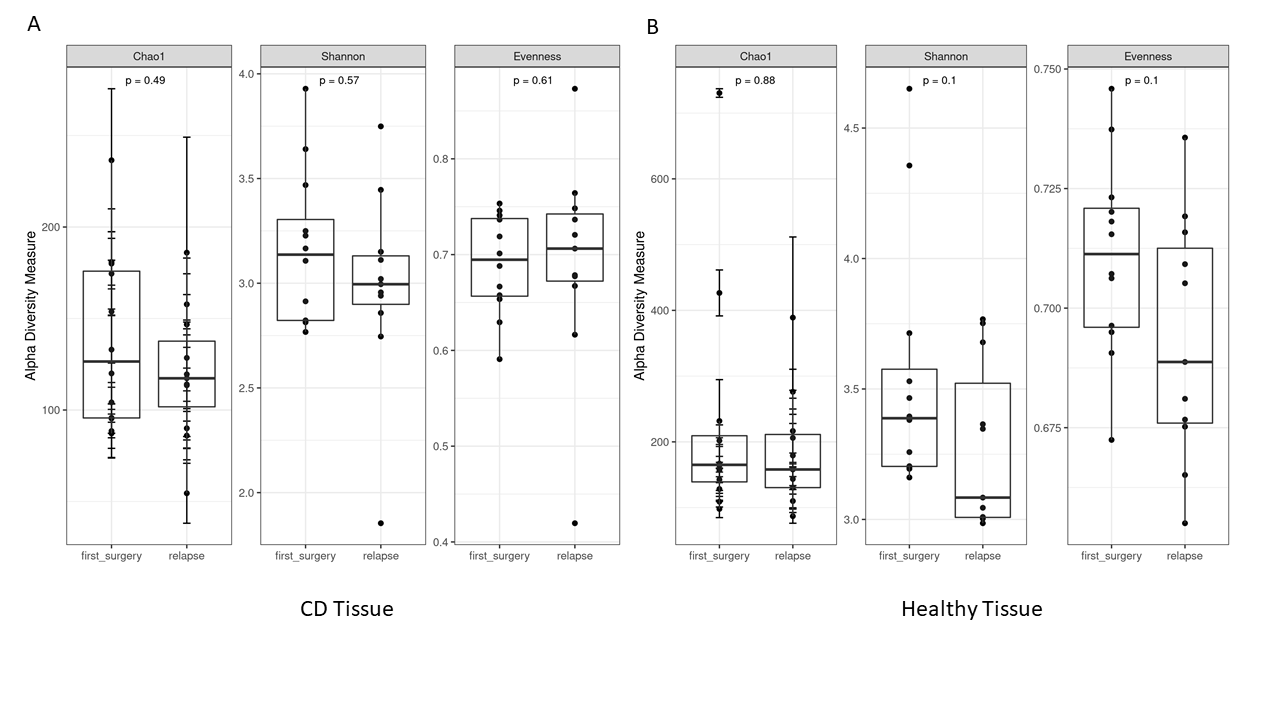

Supplement: Supplementary Figure 3 — Box-plots reporting alpha diversity indices (Chao1, Shannon and Eveness index) in first surgery and relapse patients, for both CD (A) and Healthy tissues (B). P-values less than 0.05 were considered statistically significant. [file Image_3.tif]

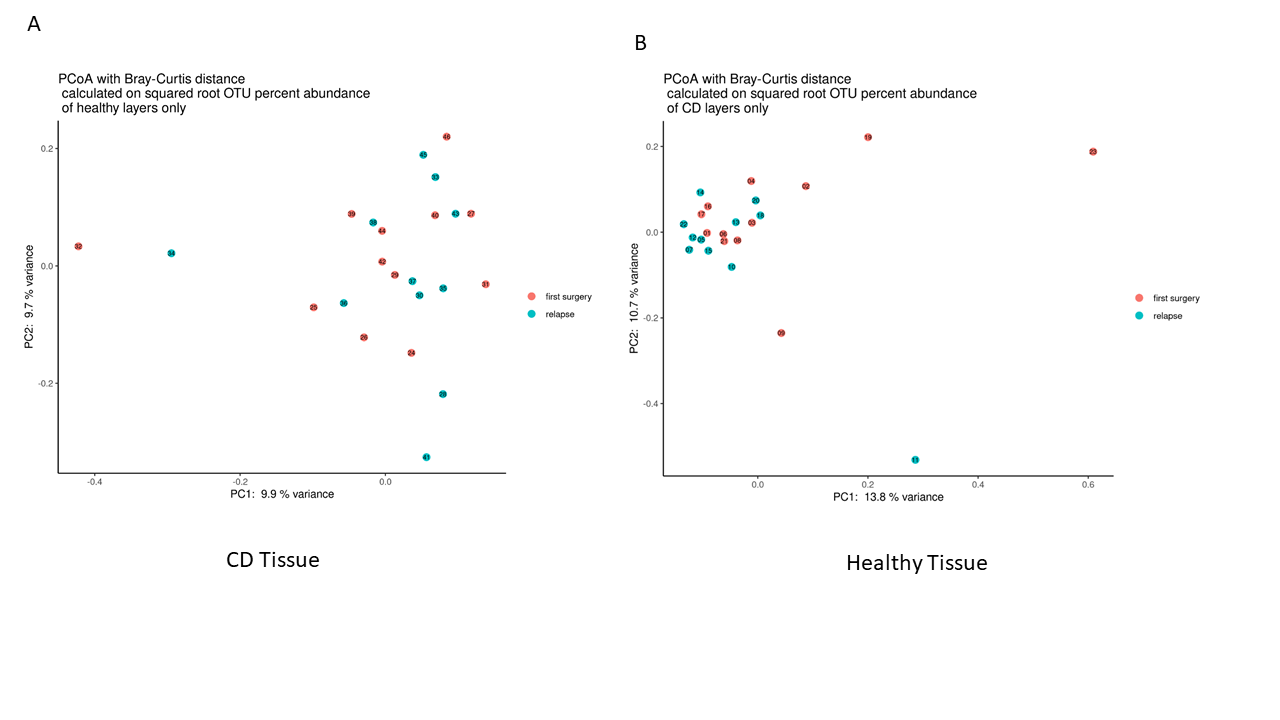

Supplement: Supplementary Figure 4 — Principal Coordinate Analysis using Bray-Curtis dissimilarity as a distance metric on square root transformed percent abundance of identified OTUs showing permuted p-value of general β dispersion and pairwise β dispersion of CD and healthy groups. The lines connect the samples from the same patient. [file Image_4.tif]
